# Comparison Between Retrograde and Antegrade Ureteroscopic Laser Lithotripsy for the Management of Medium-Sized Proximal Ureteral Stones: A Randomized Prospective Study

**DOI:** 10.7759/cureus.63196

**Published:** 2024-06-26

**Authors:** Ahmed M Abdel-Galeel, Ahmed M Abdel Gawad, Tamer A. Abouelgreed, Ahmed Y Aboelsaad, Yasser M Haggag, M Abdelwadood, Basem A Fathi, Mohamed F Elebiary, Rasha Ahmed, Ahmed G Abdel Raouf

**Affiliations:** 1 Department of Urology, Faculty of Medicine, Al-Azhar University, Cairo, EGY; 2 Department of Urology, Faculty of Medicine, Ain Shams University, Cairo, EGY

**Keywords:** laser treatment, ureteric stones, ureteroscopy, retrograde, antegrade

## Abstract

Objectives: This study aimed to present the outcomes of retrograde and antegrade ureteroscopic laser lithotripsy in the treatment of proximal ureteral stones ranging in size from 10 to 20 millimeters in diameter.

Patients and methods: From March 2023 to December 2023, 70 patients were included in this prospective randomized double-arm interventional study. Patients were divided into two groups: Group 1 (35 patients) had semi-rigid retrograde ureteroscopic laser lithotripsy, and Group 2 (35 patients) had semi-rigid antegrade ureteroscopic laser lithotripsy.

Results: In terms of length of hospitalization, there was a statistically significant distinction between the groups that were evaluated (p = 0.001). Group (1) showed a statistically significant distinction in Hb and HCT levels before and after the procedure (p < 0.05), whereas Group (2) showed a similar difference in Hb, creatinine, and HCT levels before and after the operation (p < 0.05). The antegrade group had much more hemorrhage than the retrograde group. Reduced hemoglobin (p = 0.008) and hemoglobin saturation (p = 0.029) were most noticeable in the antegrade group. Regarding stone-free rates (SFRs), no statistically significant difference was noted between the groups (p = 0.643).

Conclusion: Both retrograde and antegrade ureteroscopic laser lithotripsy are dependable and successful for the treatment of proximal ureteral stones. For medium-sized proximal ureteral stones (10-20 mm), retrograde ureteroscopic laser lithotripsy may be the first option due to its shorter hospital stays, decreased bleeding rates, blood transfusion needs, and temporary rise in serum creatinine.

## Introduction

Urolithiasis is a common health issue, with prevalence rates differing globally, spanning from 1% to 20%. They mostly involve the upper urinary tract, including ureteral and renal stones [1].

Traditionally, extracorporeal shock wave lithotripsy, ureteroscopic lithotripsy, and percutaneous nephrolithotomy are common surgical treatment options for upper urinary tract stones, with open surgery being required only in rare circumstances. Recently, advancements in technology have introduced new methods, such as flexible ureteroscopy, minimally invasive percutaneous nephrolithotomy, and laparoscopic ureterolithotomy, providing urologists with additional treatment choices [2].

Proximal ureteral stones are defined as stones located between the upper border of the L5 vertebral body and the ureteropelvic junction. They may result in pain, urinary tract infection, hydronephrosis, and even loss of function in the affected renal unit. Thus, it is necessary to find a suitable management protocol for relieving obstruction and removing stones simultaneously [3].

Although the most available treatments have been used to treat proximal ureteral stones, the optimal choice of treatment remains controversial. Extracorporeal shock wave lithotripsy has been proven to be less efficient than ureteroscopic lithotripsy in the treatment of proximal ureteral stones, particularly when dealing with large stones and critical renal insufficiency. Concerning invasiveness, laparoscopic ureterolithotomy still remains a second-line and remedial measure for other operations. Thus, ureteroscopic lithotripsy, whether retrograde or antegrade via percutaneous nephrolithotomy, is a feasible alternative for the treatment of proximal ureteral stones [1,4].

To determine which method is more effective and safer for the treatment of medium-sized proximal ureteral stones (10-20 mm), this research compared the outcomes of retrograde and antegrade ureteroscopic laser lithotripsy.

## Materials and methods

This prospective randomized double-arm interventional study was conducted with 70 patients at Al-Azhar University Hospital, New Damietta, Egypt, from March 2023 to December 2023. Patients were randomly divided into two groups using the closed envelope method: Group 1 consisted of 35 patients who underwent semirigid retrograde ureteroscopic laser lithotripsy, while Group 2 comprised 35 patients who underwent semirigid antegrade ureteroscopic laser lithotripsy.

Inclusion criteria

Patients with medium-sized proximal ureteral stones (10-20 mm) situated between the ureteropelvic junction and the top edge of the L5 vertebral body, aged 18-65 years, were included.

Exclusion criteria

Pregnancy, bleeding disorders, morbid obesity (BMI > 35), patients with stenosis of the ureter, history of previous surgery on the ipsilateral ureter, patients with a simultaneous kidney stone requiring surgery, and patients with an active urinary tract infection were excluded.

All patients were subjected to the following preoperative evaluation: clinical examination, including full history and physical examination; laboratory investigations, including complete blood count (CBC), blood group (ABO), renal function tests, liver function tests, coagulation profile, fasting blood sugar and HbA1C if diabetic, and urinalysis with culture and sensitivity testing; radiological examination, including stone characteristics evaluated by abdominopelvic ultrasound scan (USS); and computed tomography of the urinary tract (CT-UT). For patients over 40 years old, a cardiopulmonary consultation with an electrocardiogram (ECG) and a chest X-ray in addition to an echocardiography for patients over the age of 60 years.

Ethical approval

Protocols and written informed consent for all participants were approved by the Research Ethics Committee of Al-Azhar Faculty of Medicine, Nasr City, Cairo, Egypt, under IRB No. Urosurg./Ms/2023/0001 and Clinical Trial Registration No. NCT06465784.

Surgical procedure

The type of anesthesia used was spinal anesthesia.

Group 1

Group 1 included patients who had semirigid retrograde ureteroscopic laser lithotripsy.

Patient positioning: Patients were placed in the dorsal lithotomy or modified dorsal lithotomy position in which the ipsilateral leg of the ureter being examined is slightly extended and abducted to minimize the angle of the ureter over the psoas muscle and therefore facilitate the passage of the semirigid ureteroscope into the ureteral orifice and proximally into the ureter. Fluoroscopy was used with apron protection for the surgeon, nurse, and anesthetic doctor.

Operative technique: After diagnostic cystourethroscopy (Karl Storz, Germany, sheath, 22 Fr), retrograde access to the upper urinary tract was obtained by semirigid ureteroscopy (Karl Storz, Germany, 6.5/9.5 Fr) under fluoroscopy guidance. When the stone was reached, a guide wire was passed under vision to prevent stone migration. If the guide wire could not pass under vision, the stone was directly attacked at its center under fluid irrigation by normal saline using a Ho:YAG laser (Lisa Revolex DUO laser system, 2 microns) and fiber (size 272 μm). The laser energy was set at 0.8 to 1.9 joules per pulse, and the frequency was between 8 and 12 Hz. The energy and frequency could be changed during the operation according to the stone hardness and efficacy of lithotripsy. The fragmentation procedure was continued until all stone fragments were less than 4 mm. Retrograde studies with contrast medium were conducted to determine extravasation. A JJ stent was applied, and a Foley urethral catheter was inserted in all patients.

Group 2

Group 2 included patients who had semirigid antegrade ureteroscopic laser lithotripsy.

Patient positioning: Patients were placed initially in the dorsal lithotomy position and then rotated to the prone position. Fluoroscopy was used with apron protection for the surgeon, nurse, and anesthetic doctor.

Operative technique: In the lithotomy position, diagnostic cystourethroscopy (Karl Storz, Germany, sheath, 22 Fr) was performed. Retrograde access to the upper urinary tract was achieved using semirigid ureteroscopy (Karl Storz, Germany, 6.5/9.5 Fr) guided by fluoroscopy. Upon reaching the stone, a 5 Fr open-tipped ureteral catheter was inserted under direct vision to prevent stone migration. Dye was then injected to opacify the pelvicalyceal system. The distal end of the ureteral catheter was fixed to a 14 Fr Foley urethral catheter. All patients were then rotated to the prone position. When the stone was reached and the injected dye through the ureteral catheter did not reach or opacify the pelvicalyceal system, an ultrasound-guided puncture by an atraumatic Shipa needle was performed, and the dye was injected through it. Under fluoroscopic guidance, an upper or middle calyceal puncture was made with an 18-gauge puncture needle. When the needle was safely positioned in the collecting system, a J tip 0.038-inch guide wire was introduced through the needle into the pelvicalyceal system and across the ureteropelvic junction into the ureter. Then, an 8 Fr facial dilator was employed initially, and the caliber was increased gradually by progressive 2 Fr facial dilators along the guide wire until the percutaneous nephrostomy tract was dilated to 18 Fr. By using the minimally invasive percutaneous nephrostomy set (Karl Storz, Germany, sheath, 16.5/17.5), the semirigid ureteroscope (Karl Storz, Germany, 6.5/9.5 Fr) was inserted through the sheath to observe the stone, which was fragmented under fluid irrigation by normal saline using a Ho:YAG laser (Lisa Revolex DUO laser system, 2 micron), fiber (size 272 μm). The laser energy was set at 0.5 to 2.5 per pulse, and the frequency was between 8 and 12 Hz. The energy and frequency could be changed during the operation according to the stone hardness and efficacy of the lithotripsy. The fragmentation procedure was continued until all stone fragments were less than 4 mm. All patients were tubeless (nephrostomy tube not inserted). A JJ stent was applied by the antegrade method, and Foley urethral catheters were inserted in all patients.

Evaluation at follow-up

At the outpatient clinic, all patients were seen again one month after discharge. CT-UT was used to assess the first stone-free rate (SFR) (after one treatment session) and the need for any auxiliary procedures. Patients were deemed stone-free if all stones had been removed. Patients were considered to have clinically insignificant residual fragments (CIRFs) if the fragments were smaller than 4 mm in diameter, did not restrict the urine flow, and were not contaminated. The outpatient clinic evaluated all patients three months after discharge using CT-UT to check for postoperative ureteral stricture.

Sample size

The sample size was determined using the MedCalc statistical software, considering a two-sided confidence level of 95%, a power of 80%, and an α error of 5%. Based on the primary outcome of the SFR, which was estimated at 100.0% and 82.4% in the antegrade and retrograde groups, respectively [5], the initial calculated sample size was 62. To account for potential dropout cases during follow-up, the sample size was increased to 70 subjects.

Statistical analysis data

All patient data were fed into the computer and analyzed using IBM SPSS software package version 20.0 (released 2011, IBM SPSS Statistics for Windows, IBM Corp., Armonk, NY). Qualitative data were described using numbers and percentages. The Kolmogorov-Smirnov test was used to verify the normality of distribution. Quantitative data were described using range (minimum and maximum), mean, standard deviation, median, and interquartile range (IQR). The significance of the obtained results was judged at the 5% level. The tests used were the chi-square test, Student's t-test, Mann-Whitney test, and paired t-test.

## Results

**Figure 1:**
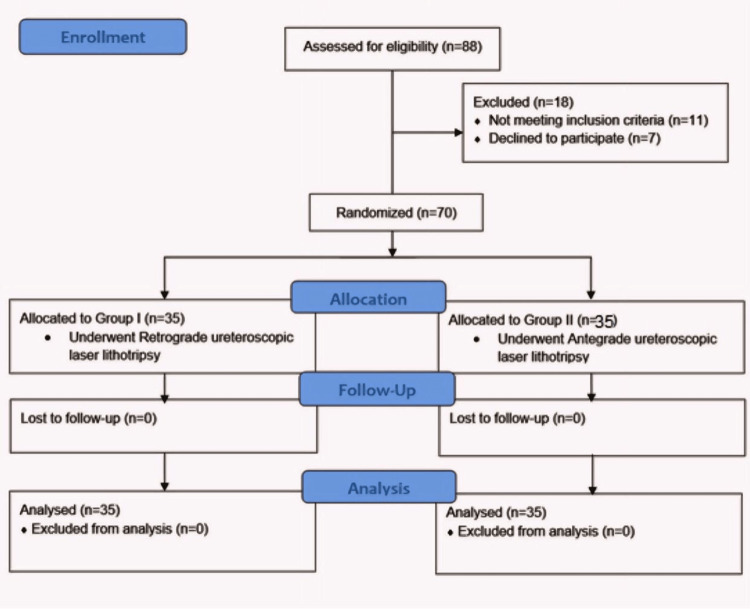
CONSORT flowchart

Eighty-eight patients were enrolled initially, but 18 were excluded. Among the exclusions, 11 patients did not meet the inclusion criteria, and seven declined to participate. Consequently, the study proceeded with 70 patients, evenly divided into two groups of 35 each (as illustrated in Figure 1).

Analysis of historical data revealed no statistically significant differences between the groups (p > 0.05; Table 1).

**Table 1:** Comparison between the studied cases according to the history data SD: standard deviation, C^2^ : Chi-square test, t: student t-test, P: p-value for comparing between studied groups, BMI: body mass index, DM: diabetes mellitus, IHD: ischemic heart disease, HTN: hypertension

Variables	Group 1 (n = 35)		Group 2 (n = 35)		Test of Sig.	P-value
Age (years)						
Range.	24–65		18–65		t = 0.842	0.403
Mean ± SD	46.2 ± 10.09		48.49 ± 12.49			
Sex	No.	%	No.	%		
Female	10	28.6	10	28.6	c^2^ = 0.0	1.0
Male	25	71.4	25	71.4		
BMI						
Range.	19–35		19–35		t = 1.437	0.155
Mean ± SD	27.13 ± 4.55		25.8 ± 3.04			
Comorbidities	No.	%	No.	%	c^2^	P
DM	7	20.0	7	20.0	0.0	1.0
IHD	2	5.7	0	0.0	2.059	0.151
HTN	7	20.0	8	22.9	0.085	0.771
Liver disease	1	2.9	0	0.0	1.014	0.314

However, when considering the severity of hydronephrosis, a statistically significant distinction was observed among the participating groups (p = 0.008; Table 2).

**Table 2:** Distribution of the studied cases according to stone data SD: standard deviation, C^2^: Chi-square test, t: Student's t-test, U: Man-Whitney test, H/U: Hounsfield unit, P: p-value for comparing between the studied groups

Variables	Group 1 (n = 35)		Group 2 (n = 35)		Test of Sig.	P-value
Laterality	No.	%	No.	%		
Left	15	42.9	17	48.6	c^2^ = 0.230	0.631
Right	20	57.1	18	51.4		
Site						
L2	7	20.0	4	11.4	c^2^ = 2.329	0.312
L3	12	34.3	18	51.4		
L4	16	45.7	13	37.1		
Ureteral stone diameter (millimeter)						
Range (mm)	10–20		10–20		t = 1.473	0.145
Mean ± SD	13.48 ± 3.2		14.69 ± 3.64			
Stone opacity	No.	%	No.	%		
Faint radio-opaque	3	8.6	2	5.7	c^2^ = 0.718	0.699
Radio-lucent	3	8.6	5	14.3		
Radio-opaque	29	82.9	28	80.0		
H/U						
Range	390–1830		466–1912		U = 582.0	0.720
Median (IQR)	1130 (890–1525)		1220 (965–1368.5)			
Degree of hydronephrosis	No.	%	No.	%		
Minimal (Grade I)	1	2.9	0	0.0	c^2 ^= 11.620	0.008
Mild (Grade II)	24	68.6	11	31.4		
Moderate (Grade III)	7	20.0	16	45.7		
Marked (Grade IV)	3	8.6	8	22.9		

Furthermore, significant differences were noted in the length of hospital stay between the evaluated groups (p = 0.001; Table 3).

**Table 3:** Comparison between studied cases according to postoperative outcome C^2^: chi-square test, P: p-value for comparing between studied groups, U: Man-Whitney test

Variables	Group 1 (n = 35)		Group 2 (n = 35)		Test of Sig.	P-value
	No.	%	No.	%	c^2^	P
Postoperative hematuria	2	5.7	5	14.3	1.429	0.232
Postoperative pain	5	14.3	9	25.7	1.429	0.232
Postoperative fever	0	0	1	2.9	1.014	0.314
Ureteral stent symptoms	5	14.3	3	8.6	0.565	0.452
Postoperative hospital stay (days)						
Range	1–2		1–3		U = 370.0	0.001
Median (IQR)	1 (1–2)		2 (1–2)			

Regarding changes in laboratory parameters, Group 1 exhibited significant variations in the Hb and HCT levels before and after the procedure (p < 0.05). Group 2 showed similar differences in Hb, creatinine, and HCT levels before and after the operation (p < 0.05). Notably, the antegrade group experienced higher rates of hemorrhage than the retrograde group, with statistically significant decreases observed in Hb (p = 0.008) and HCT (p = 0.029) levels (Table 4).

**Table 4:** Comparison between the studied cases according to perioperative laboratory investigations SD: standard deviation, t: Student's t-test, p: p-value for comparing between studied groups, HCT: hematocrit value, Hb: hemoglobin

Variables	Group 1 (n = 35)	Group 2 (n = 35)	Test of Sig.	P-value
Hb pre				
Range	10.8–15.5	11.4–15.8	t = 1.938	0.057
Mean ± SD	13.34 ± 1.14	12.8 ± 1.19		
Hb post				
Range	10.1–14.4	10.3–14.2	t = 2.733	0.008
Mean ± SD	12.6 ± 1.1	11.85 ± 1.19		
P(t1)	<0.001	<0.001		
Creatinine pre				
Range	0.45–2.05	0.45–3.2	t = 1.903	0.061
Mean ± SD	1.22 ± 0.42	1.43 ± 0.53		
Creatinine post				
Range	0.6 – 2.3	0.5–1.4	t = 0.039	0.969
Mean ± SD	1.16 ± 0.38	1.16 ± 0.19		
P(t1)	0.170	0.003		
HCT pre				
Range	33–44	32–48	t = 1.898	0.062
Mean ± SD	38.2 ± 2.77	36.65 ± 3.96		
HCT post				
Range	31.8–42	30–43	t = 2.226	0.029
Mean ± SD	36.57 ± 2.72	34.89 ± 3.52		
P(t1)	<0.001	<0.001		

Moreover, statistically significant differences were observed between the groups in terms of overall complication rates (p = 0.004) and the need for blood transfusions due to postoperative hematuria (p = 0.039; Table 5).

**Table 5:** Comparison between studied cases according to perioperative complications according to the modified Clavien-Dindo calcification P: p-value for comparing between the studied groups

Variables	Group 1 (n = 35)		Group 2 (n = 35)		Test of Sig.	P-value
	No.	%	No.	%		
Overall complications rate (number of patients who have complications)	11	31.4	23	65.7	8.235	0.004
Grade 1						
Postoperative pain	5	14.3	9	25.7	1.429	0.233
Postoperative fever	0	0.0	1	2.9	1.014	0.316
Postoperative transient rise in serum creatinine	6	17.1	11	31.4	1.942	0.164
Grade 2						
Hematuria needs a blood transfusion	0	0.0	4	11.4	4.242	0.039
Grade 3 Extravasation	2	5.7	2	5.7	0.0	1.0
Grade 4	0	0.0	0	0.0	0.0	1.0
Grade 5	0	0.0	0	0.0	0.0	1.0

Lastly, the analysis revealed no statistically significant difference between the studied groups regarding the SFR (p = 0.643; Table 6).

**Table 6:** Comparison between the studied cases according to the stone-free rate URS: ureteroscopy, PCNL: percutaneous nephrolithotripsy

Variables	Group 1 (n = 35)		Group 2 (n = 35)		Test of Sig.	P-value
	No.	%	No.	%		
Stone-free rate	32	91.4	33	94.3	0.215	0.643
Efficiency quotient	91.4100 + 2.9 + 5.7 =0.84		94.3100 + 0 + 5.7 = 0.89		0.001	0.975
Retreatment	1	2.9	0	0.0	1.014	0.316
Auxiliary procedure	2	5.7	2	5.7	0.0	1.0
Flexible URS	2	5.7	1	2.9	0.348	0.555
Mini PCNL	0	0.0	1	2.9	1.014	0.316

## Discussion

In our study, a statistically significant difference in the level of hydronephrosis was observed between the two groups under investigation (p = 0.008), aligning with the findings of Chen et al.'s study. In their research, 46 cases underwent ureteroscopic lithotripsy (URSL), while 51 cases underwent minimally invasive percutaneous nephrolithotomy (MPCNL) (p = 0.021) [6]. A potential major contributing factor to this observed difference could be the presence of case selection bias.

The two groups differed significantly in terms of the length of time they spent in the hospital (Table 3). After antegrade URS, the median duration of hospitalization was two days, but following retrograde URS, it was one day (p = 0.001). Furthermore, it was discovered by Chen et al. that the average duration of hospital stay after surgery was significantly reduced in the URSL group (1.4 ± 0.6 vs. 2.3 ± 0.7; p < 0.001) [6].

In the present study, a statistically significant difference was observed between the studied groups in terms of hemoglobin and hematocrit values before and after surgery (p < 0.001). The decrease in hemoglobin (p = 0.008) and hematocrit (p = 0.029) was notably greater in the antegrade group compared to the retrograde group. Consistent with these results, Abdeldaeim et al. reported that the mini-percutaneous nephrolithotomy group displayed a markedly higher mean postoperative reduction in hemoglobin concentration (0.47 gm vs. 0.2 gm) (p < 0.001) [7]. According to the research conducted by Chen et al., three patients from the mPCNL group required blood transfusions due to a significant drop in their hemoglobin levels [6]. Wang et al. also found that PCNL, compared to semirigid URL, increased the risk of bleeding and hematuria, often necessitating blood transfusions [8]. In alignment with these findings, four individuals in the antegrade group required blood transfusions in the present study, while none from the retrograde group needed such intervention (Table 5). No statistically significant difference between the groups in terms of postoperative pain (p = 0.233). When comparing the two methods for overall complication rates, the incidence of complications was significantly higher in the antegrade group compared to the retrograde one (p = 0.004), albeit mostly low-grade according to the Clavien classification system. This is consistent with the fact that complications like bleeding, extravasation, and transient rise in kidney functions are generally common with PCNL. This contrasts with the findings of Taguchi et al., who found no statistically significant differences in the overall complication rates between the two approaches [9].

Researchers have looked at the advantages and disadvantages of using retrograde ureteroscopy and mPNL to treat proximal ureteral stones. One study compared the two methods in 184 elderly patients with kidney and/or proximal ureteral stones measuring 10-20 mm in size. The study found that mPNL is superior for treating numerous ureteral stones, although retrograde FURL was associated with fewer problems and a shorter postoperative hospital stay [10]. Moreover, numerous studies have examined the relative merits of antegrade and retrograde URSL and drawn comparisons between the two. Zhang et al. [11] conducted a prospective, non-randomized study comparing antegrade URS (n = 32) with a retrograde approach (n = 44). Without a statistically significant difference, the authors reported SFRs of 93.7% for the antegrade group and 84.1% for the retrograde URS group [11]. After 12 weeks of follow-up, Abdeldaeim et al. [7] discovered no statistically significant difference in the SFR between the two groups (90% with flexible ureterorenoscopy and 80% with mini-percutaneous nephrolithotomy) (p = 0.472) [7].

Although there was no statistically significant difference between the two groups (p = 0.098), Chen et al. discovered that the SFR after one month in the mPCNL group was 50/51, or 98%, while in the URL group, it was 89.1% (41/46) [6]. In terms of the SFR, the present analysis indicated a statistically insignificant difference between the groups. In the antegrade group, 94.3% of stones were removed, whereas in the retrograde group, 91.4% were removed. The following description pertains to Group 1. One patient had retreatment by retrograde ureteroscopic laser lithotripsy, with a residual of 6 mm on the JJ, and retrieval was performed after six weeks after which the residual was removed by Dormia. Two patients had auxiliary procedures (flexible URS). In one of those patients, some fragments were migrated to the lower calyx, then the JJ was inserted, and flexible URS was performed after six weeks. The fragments were washed out by saline irrigation, and the laser was not used. In the other patient, two fragments about 4 mm and 5 mm were migrated to the lower calyx, then the DJ was inserted, and flexible URS was performed after six weeks.

The following description pertains to Group 2. No patient had retreatment. Two patients had auxiliary procedures (one mini-PCNL and one flexible URS). In one patient, the access to the kidney failed, so the DJ was inserted for a second trial after two weeks. On follow-up using CT-UT, the stone was found in the lower calyx, so mini-PCNL was performed. In the other patient, a residual of about 7 mm was migrated to the lower calyx, so flexible URS was used after six weeks. For proximal ureteral stones ranging in size from 10 to 20 mm, Xiao-Jian et al. found that antegrade URSL offers greater efficacy compared to retrograde URSL. At one month post-treatment, 1% of patients who underwent retrograde URSL had residual stones, while all patients in the antegrade group were stone-free at the same follow-up period [12]. Karami et al. utilized a prospective randomized study to discuss their experience with the treatment of medium-sized impacted proximal ureteral stones [13], wherein the antegrade URSL group achieved an SFR of 100%, compared to 51.4% in the retrograde URSL group. These findings are in agreement with those published by Moufid et al. and Bozkurt et al. The percentage of stones removed using the antegrade method was 95.5%, whereas the percentage using the retrograde method was 66.7% and 97.6%, respectively [14,15].

This study has some limitations. The sample was from one center. Conducting studies across various centers would enhance the robustness of the results. The follow-up duration was rather limited, spanning only three months, thus preventing us from comparing outcomes over the long term.

## Conclusions

Proximal ureteral stones, located between the upper border of the L5 vertebral body and the ureteropelvic junction, can be effectively managed with either retrograde or antegrade ureteroscopic lithotripsy via percutaneous nephrolithotomy. Our study demonstrates that both approaches are reliable and achieve effective stone clearance in the management of medium-sized proximal ureteral stones (10-20 mm). However, considering safety aspects, retrograde ureteroscopic laser lithotripsy emerges as a preferred initial choice. It is associated with shorter hospital stays, lower rates of bleeding, reduced need for blood transfusions, and a transient, manageable rise in serum creatinine levels.
